# Shopping Environment Stimuli and Their Effect on Customer Satisfaction and Purchase Loyalty in Supermarkets

**DOI:** 10.3390/foods15173149

**Published:** 2026-09-05

**Authors:** Nelson Carrión-Bósquez, Jorge Bernal-Peralta, Mónica Navarrete-Álvarez, Mario Vidal-Alfaro, Wilson Zambrano-Vélez, Ignacio López-Pastén, Paolo Cortés-Tapia, Marlon Duque-Zapata

**Affiliations:** 1 Departamento de Administración, Facultad de Economía y Administración, Universidad Católica del Norte, Antofagasta 1270709, Chile; paolo.cortes@alumnos.ucn.cl (P.C.-T.); marlon.duque@alumnos.ucn.cl (M.D.-Z.); 2 Facultad de Administración y Economía, Universidad de Tarapacá, Arica 1000007, Chile; jbernal@academicos.uta.cl (J.B.-P.); mnavarre@academicos.uta.cl (M.N.-Á.); 3 Departamento de Ingeniería Comercial, Facultad de Ingeniería, Universidad de Antofagasta, Antofagasta 1270300, Chile; mario.vidal@uantof.cl; 4 Facultad de Ciencias Sociales y de la Salud, Universidad Estatal Península de Santa Elena, La Libertad 240250, Ecuador; wzambrano@upse.edu.ec; 5 Facultad de Ingeniería y Negocios, Universidad Santo Tomás, Antofagasta 1240000, Chile; ilopez7@santotomas.cl

**Keywords:** supermarket, store design, price fairness, product variety, customer satisfaction, purchase loyalty

## Abstract

Understanding the association between supermarket environments and customer satisfaction and loyalty is important for food retailers. Based on the Stimulus–Organism–Response (SOR) model, this study examined the associations of store design, price fairness, and product variety with customer satisfaction and purchase loyalty among consumers of fast-moving consumer goods in supermarkets. Using a quantitative cross-sectional design, questionnaire data were collected from 367 supermarket customers in the Antofagasta region of Chile through non-probability sampling and analyzed using partial least squares structural equation modeling. The results of the study showed that store design (β = 0.357), price fairness (β = 0.447) and product variety (β = 0.260) were positively associated with customer satisfaction, with price fairness being the strongest environmental antecedent, while customer satisfaction showed a strong positive association with purchase loyalty (β = 0.751; *p* < 0.001) and statistically mediated the associations of store design (β = 0.268), price fairness (β = 0.336) and product variety (β = 0.195) on purchase loyalty; in addition, the R^2^ values of the model explained 51.6% of the variance in customer satisfaction and 56.3% in purchase loyalty. These findings refine the application of the SOR model in supermarket retail by showing that environmental stimuli differ in their relative explanatory strength and that customer satisfaction operates as the central mediating mechanism linking these stimuli to purchase loyalty.

## 1. Introduction

Over the past few decades, the supermarket sector has increased its relevance and has become one of the most critical links within global supply chains aimed at consumers [1]. In this regard, these establishments not only fulfill an essential role in the provision of food and basic goods but have also evolved into highly competitive environments in which firms continuously strive to attract attention and satisfy and retain their customers [2]. According to Slack et al. [3], by 2015, the global supermarket industry had reached estimated sales of USD 8.7 trillion, showing a sustained growth trend at that time. Complementarily, more recent estimates indicate that by 2023, the global retail food and grocery market will reach approximately USD 11 trillion, and industry projections suggest that this market will experience a compound annual growth rate of 3.2% over the period 2024–2030 [4]. Consistent with this international trend, Chile’s supermarket market has also demonstrated significant performance, achieving a moderate increase of 1.2%, with total sales reaching USD 18.5 billion by the end of 2025 [5].

The competitive dynamics of the supermarket sector, along with the evolution of food consumption patterns, highlight the need for continuous analysis of consumer behavior [6,7]. In this sense, supermarkets no longer depend solely on traditional strategies and are forced to understand the intrinsic and extrinsic factors that influence purchasing decisions [8], especially in emerging economies, where the growth of retail stores poses a challenge regarding the identification of consumer preferences. Therefore, identifying how customers develop loyalty towards a supermarket is a topic of interest in the academic field [9]. According to Oliver, one phase of loyalty is the conative phase. This phase is associated with the presence of repetitive episodes of behavior derived from the positive affection that an individual has for a brand [10]. Therefore, within consumption contexts, this repetitive behavioral effect is conceived as a repurchase [11], a manifestation of loyalty that can be derived from the positive levels of satisfaction experienced by a customer [12,13].

Previous studies in the retail sector have frequently examined store design (SD), price fairness (PF), and product variety (PV) separately or in non-directly comparable retail contexts, resulting in fragmented and inconsistent evidence. Graciola et al. [14] determined that there are still important gaps in the literature regarding the determinants of customer satisfaction (CS) that have not yet been covered. In this sense, the present study proposes a model that integrates variables such as SD, PF, and PV, with the purpose of examining whether these factors are associated with CS in supermarket contexts. In turn, other studies have emphasized the importance of analyzing mediating relationships of CS to deepen the understanding of purchase loyalty (PL) [15], especially in emerging economies, where consumer behavior can differ significantly from other contexts [16], which demonstrates the need to analyze the dynamics of PL in supermarkets [17]. In light of the above, the theoretical integration of Shopping Environment Stimuli allows for the comparison of their relative associations and the evaluation of their specific indirect effects through CS rather than treating the shopping environment as a homogeneous stimulus. Therefore, this integrative approach addresses the identified gap and provides context-specific evidence from an underrepresented supermarket in northern Chile. It is necessary to emphasize that in this study, PL refers to a self-reported loyalty orientation comprising respondents’ stated intention to return to the supermarket, concentration of their shopping at that establishment, and first-choice preference. Therefore, PL does not represent repeat purchasing, which is independently verified through transactional records or direct behavioral observations.

To address these knowledge gaps, this study proposes a research model that aims to identify the relationship between stimuli in a shopping environment and CS and PL in supermarkets. To consolidate the previously determined objective, this study posed the following research questions: RQ1. How do SD, PF, and PV relate to CS in supermarkets? RQ2. What relationship does CS have with PL in supermarkets? RQ3. How does CS mediate the relationship between SD, PF, and PV and PL in supermarkets? Accordingly, the theoretical contribution of this study does not rest on proposing previously unexplored relationships. Instead, it refines the stimulus component of the SOR model by integrating physical, transactional, and assortment-related stimuli, and comparing their relative associations with CS within the same model. It also examines CS as the common organismic state involved in the specific indirect paths linking these stimuli to the PL. Finally, its application in Northern Chile provides context-specific evidence regarding the applicability of the SOR model in an underrepresented emerging market supermarket setting.

## 2. Literature Review

### 2.1. Stimulus, Organism, and Response (SOR) Theory

The Stimulus–Organism–Response (SOR) model is one of the most widely used theoretical frameworks for explaining how environmental stimuli influence consumers’ internal states and, subsequently, their behavioral responses [17,18]. Several studies have employed this model as their preferred theoretical basis for consumer behavior research, using it in areas such as tourism, education, virtual reality, social responsibility, supermarket shopping, and organic consumption [19,20,21,22,23,24]. According to this approach, stimuli refer to external environmental factors that influence individuals’ perceptions and evaluations [25], demonstrating that in supermarkets, these stimuli can be associated with various elements of the shopping environment that can shape the consumer experience [26]. In the present study, these stimuli are represented by the characteristics of the supermarket environment, such as SD, PF, and PV, which can influence how consumers perceive and evaluate their shopping experience.

### 2.2. Store Design

SD refers to the set of tangible and intangible elements that shape the physical environment in which the shopping experience takes place, including aspects such as architecture, lighting, aromas, colors, and spatial layout, all of which influence how consumers perceive the service and evaluate the brand [12,27]. In the context of mass consumer goods retail, particularly in supermarkets, SD plays a strategic role by contributing to the construction of a coherent brand identity and generation of more attractive shopping experiences for consumers [14]. From the perspective of environmental psychology, various studies maintain that the characteristics of the physical environment directly affect the emotional and cognitive states of customers, impacting their behavior at the point of sale and their overall evaluation of the service [12,28].

Previous research has shown that a well-designed store can enhance CS, strengthen the perceived value of the service, and contribute to the development of longer-lasting relationships with the company [14,16]. However, the literature also reports conflicting results, as some studies have not found significant effects between the tangible elements of the store environment and CS [29,30]. These discrepancies highlight the need for further research on the influence of SD within supermarket contexts, a point supported by Faria et al. [27], who stated that more research is needed to gain a deeper understanding of the connection between SD and CS. This is also supported by Estevez et al. [31], who determined that SD is an under-emphasized variable in the academic literature. Considering the above, this study proposes the following hypotheses:

**H1.**  *SD is positively associated with CS in supermarkets*.

### 2.3. Price Fairness

PF refers to consumers’ perceptions of whether the price paid for a product or service is reasonable, fair, or consistent with the benefits received [32]. In this sense, PF is not limited solely to an economic evaluation of the price but also involves a comparative assessment between the cost incurred and the perceived benefits relative to what the consumer expects to receive [33]. The literature indicates that consumers tend to evaluate PF by considering the relationship between the economic value disbursed and the perceived performance or quality of the acquired product or service [3]. Indeed, the relevance of PF in the supermarket context lies in its level of influence on consumer behavior, as the perception of prices is a determining factor in shaping purchasing decisions and in the overall evaluation of the consumption experience [32,33,34].

Several studies have shown that the perception of PF can significantly influence CS by generating positive emotional responses associated with favorable evaluations of commercial transaction [35,36,37,38,39,40]. However, some studies have reported divergent results, indicating that certain components associated with functional value or price sensitivity do not always have significant effects on CS in everyday product purchasing contexts such as supermarkets [3,21,41]. These discrepancies highlight the need for further investigation of this relationship in less complex, everyday shopping environments, where consumption decisions tend to be more frequent and routine. Consequently, we propose the following hypothesis:

**H2.**  *PF is positively associated with CS in supermarkets*.

### 2.4. Product Variety

PV, also known as assortment variety, refers to the breadth and depth of the range of products that a supermarket offers to consumers [42]. This includes the number of categories, brands, presentations, and alternatives available to satisfy different purchasing needs [43,44]. In the retail context, this variable is also associated with the quantity and type of products that stores stock and display, as well as inventory availability, which allows them to offer multiple options to consumers [45,46]. From this perspective, greater PV makes it easier for customers to find alternatives that fit their preferences, budget, or specific requirements, which can positively influence their evaluation of the shopping experience, and consequently, their level of satisfaction [47,48].

Several studies have shown that PV is a significant factor in consumers’ perception of the value they attribute to a store, influencing both their choice of location and overall satisfaction with the service received [43,49]. Similarly, previous studies have demonstrated that a wide range of products can increase the likelihood of repeat purchases, as consumers perceive that the available assortment better meets their purchasing needs [44,50]. Furthermore, Chatzoglou et al. [12] noted that PV can directly influence CS and repurchase intention. However, other studies have reported divergent results, indicating that assortment breadth does not always have a significant effect on CS, particularly in e-commerce contexts [45,51,52]. These discrepancies highlight that the influence of PV can vary depending on the consumption context and the characteristics of the market analyzed. Therefore, we propose the following hypothesis:

**H3.**  *PV is positively associated with CS in supermarkets*.

### 2.5. Customer Satisfaction

CS refers to the psychological evaluation that arises from comparing a customer’s prior expectations with the actual performance perceived after consuming a product or service [53]. From a broader perspective, this evaluation is not limited to a single purchase experience, but is built cumulatively from multiple interactions with the establishment, allowing the consumer to develop a comprehensive assessment of the service received [2]. In this sense, CS is not restricted solely to the moment of purchase, but is also influenced by the services experienced before, during, and after the acquisition process [12]. In this sense, CS has become a strategic indicator for organizations, as it is a fundamental element for strengthening the consumer–company relationship, facilitating future transactions, and promoting favorable behaviors toward a brand [13,53,54].

In the retail sector, CS is considered a key indicator of organizational performance and the effectiveness of commercial strategies implemented by supermarkets [2]. Consequently, several studies have shown that CS acts as a relevant mediating mechanism between shopping environment attributes and consumer behavioral responses [12,55,56,57]. Recent research has highlighted that CS plays a key role in building favorable perceptions of a brand and strengthening long-term relationships between consumers and companies in retail and digital environments [16,58]. However, the literature on the relationship between CS and PL in emerging economies is scarce. Therefore, the following hypothesis is proposed:

**H4.**  *CS is positively associated with PL in supermarkets*.

Furthermore, from a consumer behavior perspective, CS contributes to strengthening consumers’ perceptions of a brand [59,60]. This demonstrates that when consumers experience positive perceptions of a company, these satisfying experiences tend to solidify favorable associations in their minds, which can reinforce how the brand is perceived and positioned against other market alternatives [61,62]. In the context of supermarkets, where shopping interactions are frequent and repetitive, accumulated satisfaction derived from positive experiences can play an important role in building more stable relationships with consumers [63,64]. Therefore, CS can act as a key psychological mechanism connecting the shopping experience with strategic outcomes, such as PL and favorable brand perceptions. In light of the above, the study tested the following hypotheses:

**H4a.**  *CS mediates the relationship between SD and PL*.

**H4b.**  *CS mediates the relationship between PF and PL*.

**H4c.**  *CS mediates the relationship between PV and PL*.

### 2.6. Research Model

**Figure 1:**
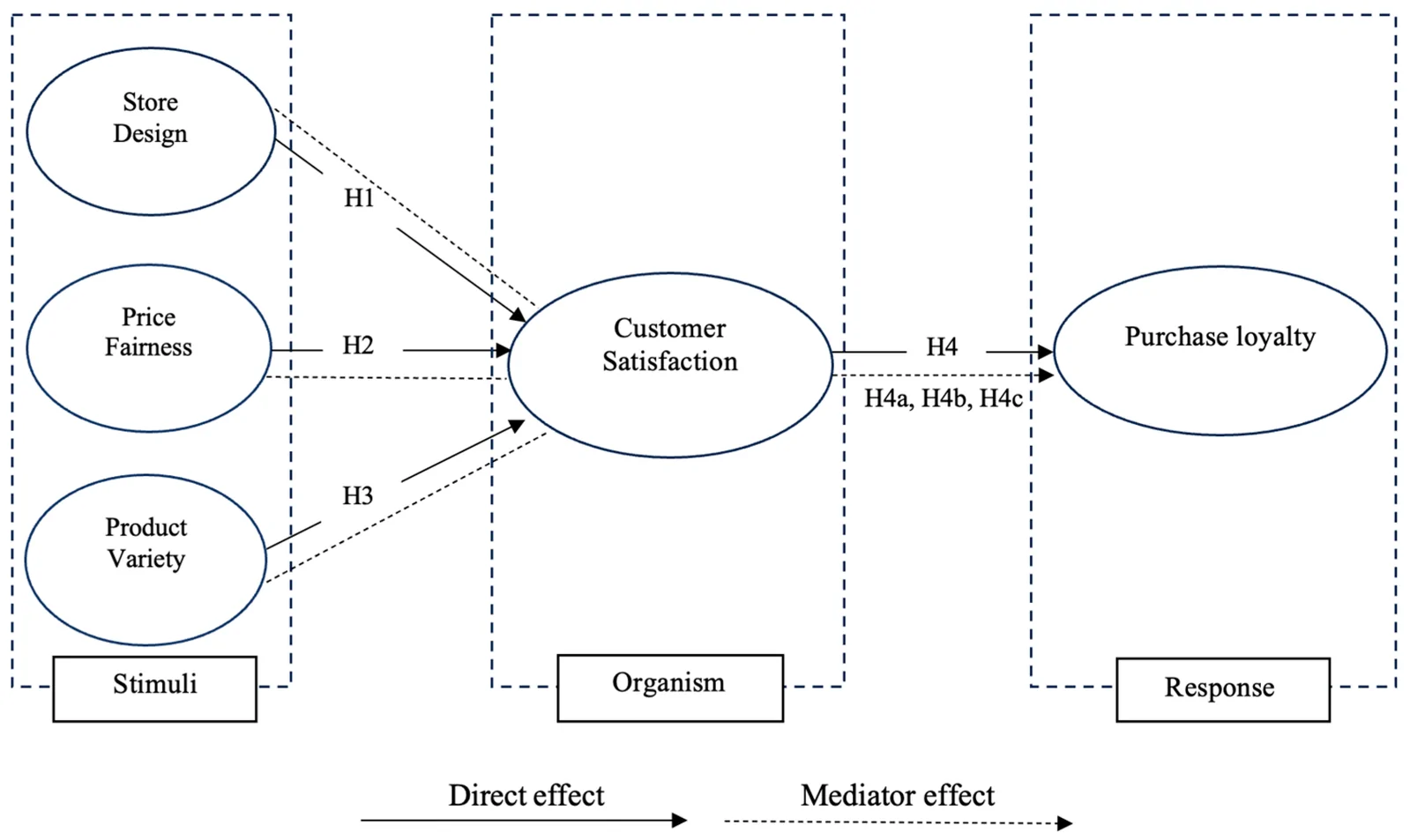
Hypothesized research model.

To facilitate an understanding of the hypotheses to be tested in this study, the research model is presented below (See Figure 1).

## 3. Methodology

### 3.1. Instrument Design and Data Collection

This study was based on a positivist paradigm. This study employed a quantitative, correlational, and cross-sectional design to measure and verify the validity of the hypothesized model. This type of research allowed for an analysis of the relationships between the variables that comprised the model at a single point in time. A survey was designed to collect information, consisting of five sociodemographic questions and 20 questions to measure the variables (four questions for each variable). The questions used to measure the study variables were adapted from previous studies published in high-impact academic journals. Specifically, the four questions to measure the SD variable were adapted from Graciola et al. [14]; the four questions to measure the PF variable were adapted from Graciola et al. [65]; and the four questions to measure the PV variable were adapted from Prediger et al. [66]. Likewise, the four questions used to measure CS were adapted from Demirci and Kara [67], whereas the four questions used to measure PL were adapted from Graciola et al. [65] (see Table A1). Responses to the questions were measured using a 7-point Likert scale, where 1 represented strongly disagree and 7 represented strongly agree.

**Figure A1:**
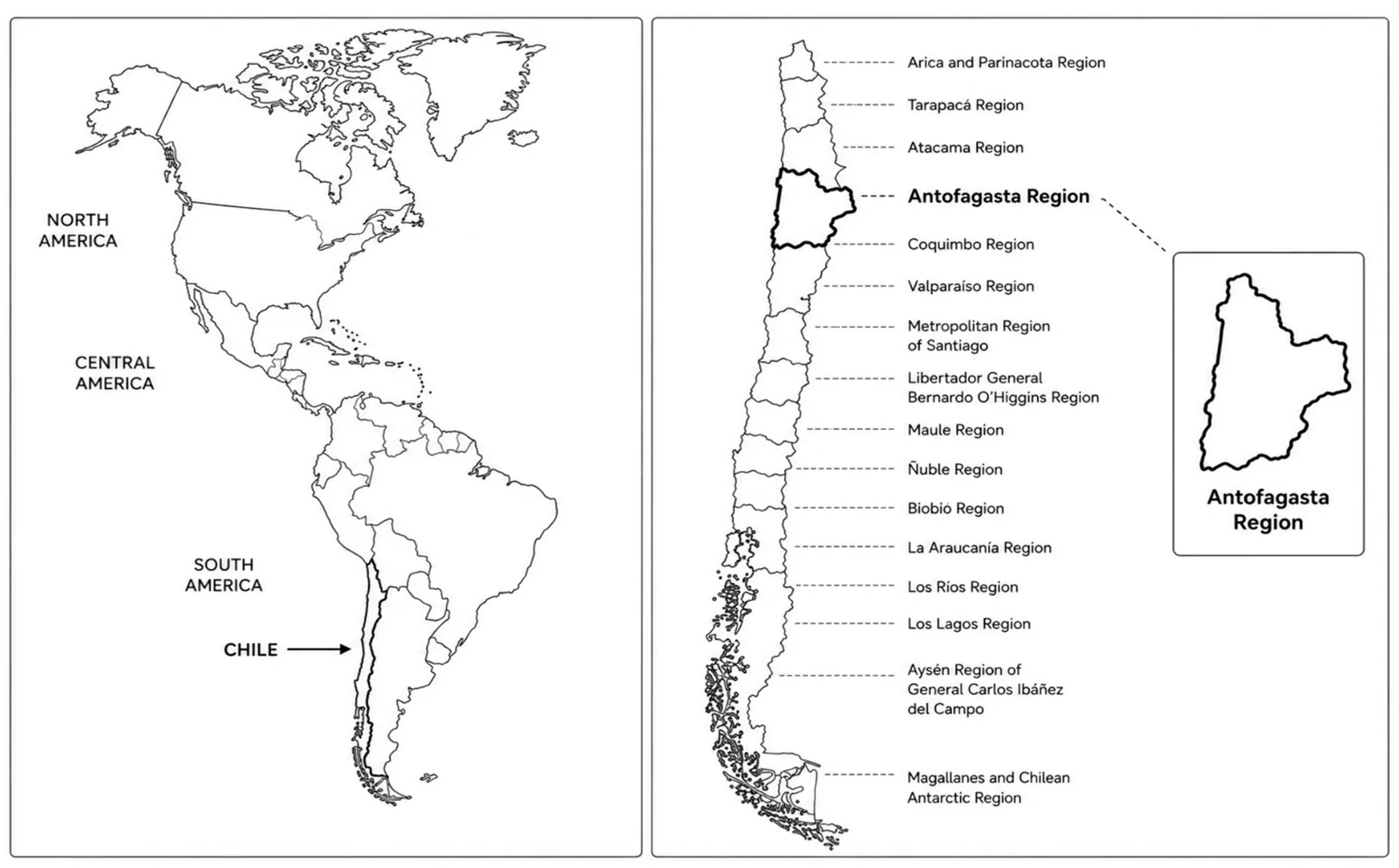
Study region.

Prior to administering the survey, the questionnaire content was validated by a panel of experts, consisting of two research specialists and two marketing specialists. No objections were received regarding the instrument. Subsequently, the functionality of the questionnaire was analyzed through a pilot test administered to 30 individuals who met the inclusion criteria for the study segment. The test participants did not receive any objections to the questionnaire. The study sample consisted of 367 mass-market consumer goods in the Antofagasta region of Chile (See Figure A1). Sample-size adequacy was assessed using G*Power 3.1, considering the maximum number of predictors of an endogenous construct (three), a medium effect size (f^2^ = 0.15), α = 0.05, and a statistical power of 0.95. The minimum required sample size was 119 participants; therefore, the final sample size of 367 participants exceeded this requirement. The inclusion criteria required that participants be supermarket customers who shopped at the same supermarket regularly (at least once a month). Using non-probability intercept sampling, respondents were recruited outside multiple supermarket stores in the Antofagasta Region. Because several participants could have been recruited at the same location, multiple respondents may have evaluated the same supermarket. The questionnaire did not record an individual store identifier; therefore, the number of respondents associated with each store could not be determined, and possible within-store clustering was not statistically accounted for. Consequently, the analysis treated individual responses as independent observations. Data collection took place in October and November 2025. To ensure that the respondents belonged to the target segment, two filter questions were used to verify whether they were frequent customers of the supermarket and whether they had recently purchased mass-market consumer goods.

### 3.2. Statistical Procedure

Given the cross-sectional and self-reported nature of the data, potential common method bias (CMB) was examined using Harman’s single-factor test and collinearity assessment based on Variance Inflation Factor (VIF) values. These procedures were employed as diagnostic checks, with values below the recommended thresholds interpreted as indicating that no substantial evidence of CMB was detected under the applied criteria, rather than confirming its complete absence [68]. Following the guidelines established by Leguina [69], the study was conducted in the following two stages: In the first stage, the measurement model is evaluated to determine the reliability and validity of the constructs. Convergent validity was examined using internal consistency tests such as standardized factor loadings, Cronbach’s alpha, and Rho_a, while reliability was tested using the Rho_c test, and construct variability was assessed using the Average Variance Extracted (AVE). Subsequently, discriminant validity was examined using two complementary criteria: the Fornell–Larcker criterion and the heterotrait–monotrait ratio (HTMT). In the second stage, the structural model was evaluated using SmartPLS 4.1.1.8 to analyze its explanatory power and test the proposed hypotheses. The statistical significance of the hypothesized relationships was determined using a non-parametric bootstrapping procedure. To interpret the results, standardized path coefficients (β), which represent the direction and magnitude of the relationships between the constructs, were examined, along with their respective t and *p*-values. Finally, the approximate fit and explanatory power of the structural model were evaluated using the Standardized Root Mean Square Residual (SRMR), coefficients of determination (R^2^), and Normalized Fit Index (NFI).

## 4. Results

### 4.1. Demographic Results

The sample consisted of 367 participants, characterized by a predominantly young profile (46.1% under 30 years old) and a slightly female majority (51.5%). Most participants reported low to middle income, primarily between one and three minimum wages (63.2%). Regarding their consumption habits, the most frequent purchase frequency was every two weeks to once a week (65.7%), with most allocating a monthly budget of between 100,000 and 600,000 Chilean pesos (70.0%). See Table 1.

### 4.2. Evaluation of Common Method Bias and Collinearity

Potential common method bias and collinearity were examined before evaluation of the measurement model. Harman’s single-factor test showed that the first unrotated factor accounted for 33.55% of the total variance, which is below the recommended threshold [70]. Moreover, the VIF values ranged from 2.110 to 2.893, which was below the threshold of 3.3 [68]. Therefore, the applied procedures did not provide substantial evidence of a common method bias or problematic collinearity. Nevertheless, these diagnostic results cannot establish the complete absence of common method bias and should be interpreted with caution.

### 4.3. Evaluation of the Measurement Model (Convergent and Discriminant Validity)

The convergent validity and reliability of the measurement model were evaluated using the SmartPLS 4.1.1.8 software. First, the internal consistency was verified. In this regard, it was found that the factor loadings of the items, Cronbach’s alpha tests, and Rho_a test reached values greater than 0.7. Furthermore, it was shown that the values obtained in the Rho_c test were greater than 0.7, and it was also verified that the values of the AVE were greater than 0.5 and less than the values of the Rho_c test. Considering the above and based on what has been stated in the literature [71,72], the existence of convergent validity of the hypothesized model was verified (See Table 2).

Discriminant validity was assessed using the HTMT index, adopting 0.90 as a maximum acceptable threshold [73]. Additionally, the Fornell–Larcker criterion [74] was evaluated, which requires the square root of the extracted mean variance to exceed the correlation values with all other constructs in the model. As shown in Table 3, all HTMT coefficients are below 0.900, and all SRAVE values on the diagonal are greater than the correlations between the constructs. Taken together, these tests confirmed satisfactory discriminant validity (Table 3).

### 4.4. Evaluation of the Structural Model (Model Fitting and Hypothesis Testing)

In accordance with the conceptual sequence proposed by the S-O-R model, the structural model was specified to examine the associations in the stimulus–organism–response sequence. Therefore, SD, PF, and PV represent the environmental stimuli associated with CS as an evaluative state of the organism, which in turn is associated with PL as a response. In this sense, the evaluation of the structural model does not include explanations of the direct routes from these environmental stimuli (SD, PF, PV) to PL, as the central purpose of the study was to evaluate the specific indirect effects that operate through CS within the proposed SOR sequence.

The structural model was analyzed using SmartPLS 4.1.1.8, and the significance of the proposed relationships and structural paths of the hypotheses were evaluated through a bootstrapping procedure (5000 samples) [75]. The R^2^ coefficients of the endogenous variables were analyzed to assess the predictive capacity of the proposed relationships. In this regard, Chin [76] stated that R^2^ values greater than 0.10 are considered acceptable and demonstrate that the model has explanatory power. The results obtained are as follows: CS (R^2^ = 0.516) and PL (R^2^ = 0.563). Furthermore, to evaluate how well the theoretical model fits the observed real data, SRMR was calculated. According to Roemer et al. [77], values less than 0.1 indicate an adequate fit of the model; in this sense, the SRMR value obtained was 0.047. In addition to the indicators described above, the NFI was analyzed to determine whether the model adequately explained the covariances. Kline [78] states that when this index yields values greater than 0.90, it confirms that the theoretical model explains the covariance of the data significantly. The result obtained in this study was 0.906.

Furthermore, analysis of the relationships between the variables yielded the following results: SD (β: 0.357, *p* < 0.001), PF (β: 0.447, *p*-value: <0.001), and PV (β: 0.260, *p*-value: <0.001) were related to CS. CS (β: 0.751, *p* < 0.001) was related to PL. Furthermore, regarding the mediating relationships, the study found that CS mediates the relationship between SD and PL (β: 0.268, *p*-value: <0.001), that CS mediates the relationship between PF and PL (β: 0.336, *p*-value: <0.001), and that CS mediates the relationship between PV and PL (β: 0.195, *p*-value: <0.001). In addition, the t-statistic values of the hypothetical relationships proposed in the model were verified, showing that the values obtained for the relationships were >1.96. According to Nieminen [79], when the path coefficients β report values less than 0.30, the hypothetical relationships are weak; if they show values between 0.30 and 0.50, they are moderate; and if the values are greater than 0.50, they show strong relationships. Hair et al. [71] determined that when the critical t-statistic values were greater than 1.96, and the statistical significance was less than 0.05, the relationship between the hypotheses proposed in the model was validated. Finally, the bootstrap analysis supported the three specific indirect effects because their 95% Confidence Intervals (CI) did not include zero. Specifically, significant indirect associations were observed for SD, PF, and PV with PL through CS (See Table 4).

## 5. Discussion

To facilitate understanding of the study’s findings, a discussion of the results will be presented to answer the following research questions: RQ1. How are SD, PF, and PV associated with CS in supermarkets? RQ2. How is CS associated with self-reported PL in supermarkets? RQ3. What specific indirect associations link SD, PF, and PV with PL through CS? Given the cross-sectional and correlational design of the study, the findings discussed in the following subsections are interpreted as statistical associations rather than intervention effects. Accordingly, managerial considerations concerning SD, PF, PV, CS, and PL should be understood as plausible implications of the observed associations.

### 5.1. Relationship Between Store Design, Price Fairness, and Product Variety and Customer Satisfaction in Supermarkets

Based on the empirical analyses conducted, H1 was supported; that is, SD is related to CS in supermarkets. Conceptually, this finding suggests that a store’s configuration is a relevant component for evaluating the shopping experience. In practical terms, it highlights the importance of strategically managing architecture, lighting, colors, aromas, and spatial arrangements to enhance CS. This relationship is explained by the fact that these tangible and intangible elements shape the environment in which the service is provided and influence the customer’s emotional and cognitive states as well as their evaluation of the store [12,27,28]. This result aligns with Graciola et al. [14] and Ananda et al. [16], who maintain that appropriate design fosters satisfaction, perceived value, and the building of lasting relationships; however, this differs from Slack et al. [29] and Rashid et al. [30], who did not identify significant associations of tangible elements, suggesting that this relationship may depend on the retail context analyzed. Considering the findings derived from this hypothesis, it is proven that SD represents the external stimulus that shapes the shopping experience, whereas CS constitutes the organism, reflecting an internal psychological evaluation of perceived performance against consumer expectations. Consequently, it was confirmed that environmental stimuli modify the internal states that precede behavioral responses.

The results of the structural model support H2; therefore, it was supported that PF is related to CS in supermarkets. This finding suggests that satisfaction does not depend solely on monetary price, but rather on the consumer considering it reasonable, equitable, and consistent with the benefits obtained. In practical terms, supermarkets should strive for a perceptible correspondence between the price charged, quality offered, and value received. This relationship arises because consumers compare the cost incurred with the expected benefits and perceived performance of a product or service [3,32,33]. When this comparison is favorable, it generates positive emotional responses and a satisfactory evaluation of the transaction. This result aligns with those of Herrmann et al. [35], Chen et al. [36], Konuk [37], Ahmed et al. [38], Cárdenas et al. [39], and Sun and Moon [40], who identified PF as a significant antecedent of satisfaction. However, this differs from [3,21] and Hui et al. [41], whose findings indicate that functional value or price sensitivity does not always produce significant associations in everyday purchases. Considering the evidence, it is verified that PF constitutes an external stimulus of the commercial environment that guides the perceptions and evaluations of the consumer, whereas CS represents the organism by expressing the psychological state resulting from comparing expectations and perceived performance, confirming that a favorable commercial stimulus transforms the internal evaluative state of the consumer before generating behavioral responses.

The evidence obtained in the study supported H3, thus accepting that PV is related to supermarket CS, although its magnitude was low in relation to the variables discussed previously. This result suggests that the breadth and depth of the assortment contribute to a favorable evaluation of the shopping experience. From a managerial perspective, supermarkets could offer categories, presentations, and alternatives that are capable of responding to different preferences, budgets, and requirements. The underlying logic is that a diversified offering increases the likelihood that consumers will find products tailored to their needs, which strengthens the value attributed to the establishment and promotes their satisfaction [42,47,48]. This finding coincides with Huddleston et al. [43,49], and Chatzoglou et al. [12], who recognized that assortment diversity influences the evaluation of service, satisfaction, and repurchase intentions, and aligns with Mofokeng [44] and Kulkarni [50], who found that a broad product range better meets purchasing needs. However, this differs from Tata et al. [45], Gázquez et al. [51], and Sethuraman et al. [52], who did not find a significant relationship in e-commerce contexts, revealing that the influence of product assortments can vary depending on the consumption environment. Based on the findings presented in this hypothesis, PV acts as an external stimulus that shapes the buyer’s experience and evaluations, whereas CS represents the organism, constituting an internal psychological assessment of the experience received.

From an integrated perspective of the three shopping environment stimuli, PF showed the strongest standardized association with CS (β = 0.447), followed by SD (β = 0.357), and PV (β = 0.260). A plausible explanation for this pattern is the evaluative proximity of each stimulus to the central supermarket transaction. While SD represents an atmospheric and experiential cue and product variety reflects the functional adequacy of the assortment, PF directly captures whether consumers consider the economic sacrifice of the purchase to be reasonable in relation to the quality and benefits received [3,32,33]. Given that supermarket shopping is frequent, and prices are visible and comparable across products and stores, perceived fairness can constitute a particularly useful diagnostic basis for evaluating the overall shopping experience. This interpretation is consistent with previous studies linking favorable price evaluations with CS [35,36,37,38,39,40], whereas the mixed results reported in other contexts [3,21,41] suggest that their relevance may depend on the characteristics of the retail environment. Price-related evaluations may also have been particularly relevant in the current sample, as 63.2% of the participants reported incomes between one and three minimum wages.

### 5.2. Relationship Between Customer Satisfaction and Purchase Loyalty in Supermarkets

Based on the empirical evidence obtained, H4 was accepted; that is, it was verified that CS is related to PL in supermarkets. This finding suggests that a satisfactory evaluation of experiences with the supermarket favors the continuity of the business relationship and repeat purchases. Therefore, from a practical perspective, satisfaction constitutes a strategic asset for retaining customers and stimulating future transactions. This association is explained by the fact that satisfaction arises from the comparison between prior expectations and perceived performance, and is cumulatively consolidated through interactions experienced before, during, and after the purchase [2,12,53]. This suggests that when this evaluation is favorable, the consumer–company relationship is strengthened, and positive behaviors toward the establishment are promoted [13,54]. This finding highlights that the conative phase of loyalty manifests itself through repetitive behaviors derived from positive affect toward a brand, where repurchase becomes a behavioral manifestation associated with satisfactory experiences [10,11,12,13]. From a theoretical perspective, it is verified that CS represents an internal psychological and evaluative state of the consumer and that PL becomes the response, expressed through the continuity and repetition of purchase behavior.

### 5.3. Customer Satisfaction and Its Mediating Association with the Relationship Between Store Design, Price Fairness, Product Variety, and Purchase Loyalty in Supermarkets

The indirect relationship obtained allows us to accept H4a, identifying a positive and statistically significant specific indirect association between SD and PL through CS. This result suggests that the contribution of SD to loyalty is channeled through the satisfaction generated during the shopping experience. In practical terms, architecture, lighting, colors, aromas, and spatial arrangement should be managed as resources that are capable of fostering lasting relationships with customers. This mediation is explained by the fact that the elements of the establishment initially influence emotional and cognitive states and the overall evaluation of the service [12,27,28]. Subsequently, accumulated satisfaction strengthens the consumer–company relationship and encourages repeat business [2,63,64]. This finding aligns with Graciola et al. [14] and Ananda et al. [16], who linked appropriate design with satisfaction and longer-lasting relationships and supported the mediating role attributed to satisfaction by Zhong and Moon [55], Han et al. [56], Chatzoglou et al. [12], and Lin et al. [57]. However, this differs from the findings of Slack et al. [29] and Rashid et al. [30], who did not identify significant associations between tangible elements and satisfaction. From a theoretical perspective, SD can be positioned as the stimulus, CS as the organism, and PL as the response; therefore, the observed pattern is consistent with the proposed S → O → R sequence.

The bootstrap analysis supported H4b by identifying a positive and statistically significant specific indirect association between PF and PL through CS. Conceptually, this result suggests that the perception of reasonable equitable prices consistent with the benefits received fosters loyalty by generating a satisfactory experience beforehand. In practice, supermarkets must strive for a match between the price, quality, and value delivered to strengthen customer retention. This mediation arises because consumers compare the costs incurred with expected benefits and perceived performance [3,32,33]. When this evaluation is favorable, it generates positive emotional responses that increase satisfaction and facilitate future transactions. This finding aligns with Herrmann et al. [35], Chen et al. [36], Konuk [37], Ahmed et al. [38], Cárdenas et al. [39], and Sun and Moon [40], who linked PF to satisfaction and complemented existing research on satisfaction as a mechanism between purchase attributes and behavioral responses [55], Han et al. [56], and Lin et al. [57]. However, this contrasts with the opposing views of Slack et al. [3], Cakici and Tekeli [21], and Hui et al. [41]. Therefore, PF can be theoretically positioned as the stimulus, CS as the organism, and PL as the response, with the observed pattern being consistent with the proposed S → O → R sequence.

Finally, the indirect association obtained allowed us to accept H4c, thus confirming that CS mediates the relationship between PV and PL, as the indirect relationship was positive and statistically significant, albeit of low magnitude. This finding demonstrates that variety not only leads to a favorable evaluation of the supermarket, but can also translate into loyalty when the available assortment already satisfies consumer needs. Therefore, offering categories, presentations, and alternatives tailored to different preferences and budgets is strategic. This relationship is explained by the fact that greater diversity increases the chances of finding relevant options, raises the value attributed to the establishment, and fosters satisfaction, which subsequently strengthens repeat purchases [12,47,48]. This result is consistent with Huddleston et al. [43], Mofokeng [44], and Kulkarni [50], but differs from those of Tata et al. [45], Gázquez et al. [51], and Sethuraman et al. [52], who reported non-significant results in electronic contexts, highlighting the relevance of the consumption environment. In light of the above, PV can be theoretically positioned as the stimulus, CS as the organism, and PL as the response; consequently, the observed pattern is consistent with the proposed S → O → R sequence.

## 6. Conclusions

The study concludes that supermarket environmental stimuli are not equally associated with CS. In this sense, PF and SD showed moderate positive associations with PF emerging as the most prominent stimulus, whereas PV displayed a positive but comparatively weak association. This hierarchy suggests that within the analyzed supermarket context, consumers’ evaluations may be more closely anchored in the perceived fairness of the economic exchange than in the physical attractiveness of the store or the breadth of its assortment. Nevertheless, SD and PV remained relevant components of the overall shopping experience. CS exhibited the strongest relationship in the model, showing a strong association with PL and highlighting its central role as an organismic evaluative state within the S-O-R sequence. The specific indirect effects reflected a differentiated pattern: the indirect association involving PF was moderate, whereas that involving SD and PV was weaker. Thus, the findings refine the SOR framework by showing that its stimulus component is heterogeneous and that the relative relevance of each stimulus depends on its evaluative proximity to the supermarket exchange. However, these comparisons reflect differences in the coefficient magnitude rather than formally tested differences between paths. Moreover, the results demonstrate statistically significant indirect associations, but they do not establish complete mediation, temporal ordering, causal relationships, or objectively verified repeat purchases. Therefore, the conclusions remain specific to the non-probability sample of supermarket customers analyzed in Northern Chile.

## 7. Theoretical, Practical, and Social Implications

This study provides empirical evidence that, viewed from a theoretical, practical, and social perspective, supports decision-making within emerging economies such as Chile. From a theoretical perspective, the findings strengthen the explanatory power of the SOR model in the context of supermarkets in Northern Chile, demonstrating that environmental stimuli do not all have the same explanatory power. PF occupied a predominant position, followed by SD, and finally PV. This allows us to move beyond the homogeneous interpretation of the “stimulus” component and demonstrates that consumers establish hierarchies when evaluating the attributes of the commercial environment. Furthermore, CS acquires a dual theoretical function: it is both a result of external stimuli and the psychological mechanism that connects these stimuli with the behavioral response. Consequently, the study broadens the understanding of loyalty by demonstrating that it does not automatically emerge from commercial attributes, but rather from the favorable interpretation that consumers construct about their experience.

From a managerial perspective, the relative magnitude of the observed associations suggests that supermarket managers in Northern Chile could initially prioritize the monitoring of PF, which showed the largest association with CS and the largest specific indirect association with PL. In this sense, potential initiatives for local testing include clearer unit price information, consistency between shelf and checkout prices, transparent promotional conditions, and affordable alternatives across different price and package-size levels. These considerations may be especially relevant because 63.2% of participants reported incomes between one and three times the minimum wage. SD, which showed the second-largest association, can be evaluated through customer feedback on architecture, lighting, colors, decoration, and spatial layout. Regarding PV, the smaller but significant association suggests focusing on the relevance and availability of products across brands, categories, and PL, rather than indiscriminately expanding the assortment. Finally, given the strong association between CS and PL, managers could systematically monitor overall satisfaction, service quality, and the fulfillment of expectations.

It is important to recognize the social implications, especially since supermarkets sell essential consumer goods and their decisions directly affect household budgets. In contexts characterized by budget constraints and concerns about the cost of living, PF transcends its commercial function and takes on the dimensions of well-being, trust, and responsibility toward consumers. Therefore, offering prices perceived as equitable, alternatives corresponding to different income levels, and adequate product availability can help reduce the feeling of disadvantage during a shopping experience. Likewise, a clear, organized, and functional shopping environment can foster more inclusive experiences for consumers with diverse needs. In this way, the S-O-R Model sequence also has a social dimension: responsible business practices act as incentives that generate internal evaluations of trust and satisfaction, which can translate into more stable relationships with establishments.

## 8. Limitations and Recommendations for Future Research

Although the research results support the relationships proposed in the hypothesized model, it is necessary to point out that the study has several methodological limitations. First, the cross-sectional and correlational design prevents the establishment of causality and verifies the temporal sequence postulated by the SOR model; therefore, the term “influence” represents a structural association and not a demonstrated causal relationship. Second, non-probability sampling, limited to the Antofagasta region, restricts the generalizability of the results to other territories, supermarket formats, and socioeconomic segments. The concentration of young participants and those with middle and low incomes may also have influenced the evaluations, particularly regarding PF. Third, including only repeat customers of the same supermarket and surveying them immediately after their purchase may generate a selection bias favoring satisfaction and loyalty, excluding dissatisfied consumers, occasional shoppers, customers who switched stores, and digital channel users. Furthermore, the questionnaire did not verify whether each respondent had previously used the self-checkout system. Consequently, responses to CS4 may reflect different levels of familiarity or direct experience with this service, which limits its interpretation as an indicator of overall CS. On the other hand, the use of a self-reported questionnaire, administered at a single point in time and by a single source, maintains the risk of social desirability bias, artificial consistency, and common method bias, which reduces this concern but does not allow us to claim its complete absence. Additionally, multiple respondents may have evaluated the same supermarket; however, store identifiers were not recorded and possible within-store clustering was not statistically modeled; in this sense, the observations may not have been completely independent, and this unaccounted dependence could have affected the precision of the estimated standard errors and significance tests. In addition, loyalty was assessed perceptually and not through verified purchasing behavior. The model also restricts the organism to satisfaction and loyalty responses, leaving out other relevant psychological processes and behaviors.

To overcome these limitations, it is recommended to develop longitudinal, panel, or experimental studies that allow us to observe how modifications in prices, design, and assortment produce subsequent changes in satisfaction and loyalty, strengthening the causal interpretation of the S → O → R sequence. Probabilistic and multiregional samples incorporating different cities, socioeconomic levels, age groups, and retail formats should also be used, complemented by multigroup analyses that determine whether the relationships vary according to income, age, sex, or purchase frequency. Participant selection should include frequent and occasional customers, consumers who have switched to supermarkets, dissatisfied individuals, and shoppers from both physical and digital channels; also, future studies should record store-level identifiers and employ cluster-adjusted or multilevel analyses. To reduce self-reporting bias, it is advisable to combine surveys administered at different times with transactional records, actual purchase frequency, observed spending, and store-switching behavior. Theoretically, future research could expand the S-O-R model by incorporating additional internal states such as emotions, perceived value, and trust, as well as responses beyond declared loyalty.

## Figures and Tables

**Table 1:** Demographic description of the participants.

Variable		N	%
Age	Between 18 and 23 years old	88	24.0%
	Between 24 and 29 years old	81	22.1%
	Between 30 and 35 years old	59	16.1%
	Between 36 and 41 years old	41	11.2%
	Between 42 and 47 years old	49	13.4%
	Between 48 and 53 years old	26	7.1%
	Over 54 years old	23	6.3%
Gender	Male	169	46.0%
	Female	189	51.5%
	I prefer not to say	9	2.5%
Income (1 basic salary = 553,553 pesos)	Less than a basic salary	50	13.6%
	Between 1 and 2 basic salaries	115	31.3%
	Between 2 and 3 basic salaries	117	31.9%
	Between 3 and 4 basic incomes	54	14.7%
	Between 4 and 5 basic incomes	18	4.9%
	Greater than 5 basic incomes	13	3.5
Purchase frequency	Several times a week	21	5.7%
	Once a week	109	29.7%
	Every two weeks	132	36.0%
	Once a month	105	28.6%
Estimated shopping expenses (Monthly)	Less than 100,000 pesos	33	9%
	Between 100 thousand and 300 thousand pesos	102	27.8%
	Between 300 thousand and 600 thousand pesos	155	42.2%
	Between 600 thousand and 1 million pesos	61	16.6%
	More than 1 million pesos	16	4.4%

**Table 2:** Convergent validity of the model.

Variable		Factor Loading	Cronbach’s Alpha	Reliability		AVE
				Rho_a	Rho_c	
Store Design	SD1	0.868	0.880	0.880	0.917	0.735
	SD2	0.847				
	SD3	0.851				
	SD4	0.863				
Price Fairness	PF1	0.885	0.895	0.898	0.927	0.760
	PF2	0.841				
	PF3	0.892				
	PF4	0.869				
Product Variety	PV1	0.891	0.897	0.898	0.928	0.763
	PV2	0.859				
	PV3	0.876				
	PV4	0.869				
Customer Satisfaction	CS1	0.874	0.907	0.907	0.935	0.782
	CS2	0.871				
	CS3	0.891				
	CS4	0.900				
Purchase loyalty	PL1	0.854	0.893	0.894	0.926	0.758
	PL2	0.856				
	PL3	0.875				
	PL4	0.896				

**Table 3:** Discriminant validity.

	SD	PF	PV	CS	PL
SD	0.857	0.850	0.178	0.527	0.405
PF	0.164	0.872	0.190	0.609	0.561
PV	0.157	0.170	0.874	0.434	0.427
CS	0.471	0.550	0.392	0.884	0.833
PL	0.360	0.502	0.383	0.751	0.870

HTMT: values above the diagonal; Fornell and Larcker: values on the diagonal; correlations of the constructs: values below the diagonal.

**Table 4:** Hypothesis testing.

**Direct Effect**						
**Hypothesis**		**β**	***p*** **-Value**	**T-Statistic**		**Hypothesis**
H1	SD-CS	0.357	<0.001	10.283		Accepted
H2	PF-CS	0.447	<0.001	12.551		Accepted
H3	PV-CS	0.260	<0.001	7.164		Accepted
H4	CS-PL	0.751	<0.001	30.773		Accepted
**Indirect Effect**						
**Hypothesis**		**β**	***p*** **-Value**	**T-Statistic**	**CI [LL–UL]**	**Hypothesis**
H4a	SD → CS → PL	0.268	<0.001	9.348	[0.212, 0.325]	Accepted
H4b	PF → CS → PL	0.336	<0.001	11.484	[0.277, 0.392]	Accepted
H4c	PV → CS → PL	0.195	<0.001	6.975	[0.139, 0.251]	Accepted

SRMR: 0.047; NFI: 0.906; R^2^CS: 0.516; R^2^PL: 0.563. CI: confidence interval; LL: 2.5% lower limit; UL: 97.5% upper limit.

## Data Availability

The data presented in this study are openly available in Google Drive at https://drive.google.com/drive/folders/1jdLFayQPFbxi-3T3xbMdGwwps6TZrCsZ?usp=drive_link (accessed on 2 August 2026).

## Abbreviations

The following abbreviations are used in this manuscript:

SD	Store Design
PF	Price Fairness
PV	Product Variety
CS	Customer Satisfaction
PL	Purchase Loyalty
AVE	Average Variance Extracted
HTMT	Heterotrait–Monotrait Ratio
SRMR	Standardized Root Mean Square Residual
NFI	Normalized Fit Index

## Appendix A

**Table A1:** Survey questions.

Variable		Question	Author
Store Design	SD1	The architecture of this supermarket is appealing.	[14]
	SD2	The supermarket is attractively decorated.	
	SD3	The color palette of the interior walls and floors is attractive.	
	SD4	The overall design of this supermarket is interesting.	
Price Fairness	PF1	This supermarket offers products at a fair price.	[65]
	PF2	This supermarket offers products at an acceptable price.	
	PF3	The price of products in this supermarket is justifiable.	
	PF4	This supermarket offers products at reasonable prices.	
Product Variety	PV1	This supermarket has a wide variety of products.	[66]
	PV2	It seems I have everything I need at this supermarket.	
	PV3	This supermarket seems to have a wide variety of brands.	
	PV4	This supermarket has a great selection of products.	
Customer Satisfaction	CS1	Overall, I am very satisfied with this supermarket.	[67]
	CS2	I am extremely satisfied with the quality of service at this supermarket.	
	CS3	This supermarket meets my expectations.	
	CS4	I am extremely satisfied with the quality of service at the self-checkout system.	
Purchase Loyalty	PL1	If I go shopping today, I will go back to this supermarket.	[65]
	PL2	I do most of my shopping at this supermarket.	
	PL3	When I go shopping, I consider this supermarket first.	
	PL4	When I go shopping, this supermarket is my first choice.	
